# Machine-Learning Assisted Optimisation of Free-Parameters of a Dual-Input Power Amplifier for Wideband Applications

**DOI:** 10.3390/s21082831

**Published:** 2021-04-17

**Authors:** Teng Wang, Wantao Li, Roberto Quaglia, Pere L. Gilabert

**Affiliations:** 1 Department of Signal Theory and Communications, Universitat Politècnica de Catalunya (UPC)—Barcelona Tech, 08034 Barcelona, Spain; teng.wang@estudiant.upc.edu (T.W.); wantao.li@upc.edu (W.L.); 2 Centre for High Frequency Engineering, Cardiff University, Queen’s Buildings, The Parade, Cardiff CF24 3AA, UK; quagliar@cardiff.ac.uk

**Keywords:** digital predistortion, global optimisation, load modulated power amplifier

## Abstract

This paper presents an auto-tuning approach for dual-input power amplifiers using a combination of global optimisation search algorithms and adaptive linearisation in the optimisation of a multiple-input power amplifier. The objective is to exploit the extra degrees of freedom provided by dual-input topologies to enhance the power efficiency figures along wide signal bandwidths and high peak-to-average power ratio values, while being compliant with the linearity requirements. By using heuristic search global optimisation algorithms, such as the simulated annealing or the adaptive Lipschitz Optimisation, it is possible to find the best parameter configuration for PA biasing, signal calibration, and digital predistortion linearisation to help mitigating the inherent trade-off between linearity and power efficiency. Experimental results using a load-modulated balanced amplifier as device-under-test showed that after properly tuning the selected free-parameters it was possible to maximise the power efficiency when considering long-term evolution signals with different bandwidths. For example, a carrier aggregated a long-term evolution signal with up to 200 MHz instantaneous bandwidth and a peak-to-average power ratio greater than 10 dB, and was amplified with a mean output power around 33 dBm and 22.2% of mean power efficiency while meeting the in-band (error vector magnitude lower than 1%) and out-of-band (adjacent channel leakage ratio lower than −45 dBc) linearity requirements.

## 1. Introduction

Current and future generations of mobile communications are not only oriented at satisfying human communication in the form of voice, data, and Internet, but also aim for industrial communications capable to foster the transformation into a global digital and sustainable economy. One unstoppable trend is the always increasing demand for high data rates (and low latency) to serve the continuously growing number of users and volume of data traffic. Over the past years, several efforts have been devoted to increase the spectral efficiency in wireless communications (e.g., multi-carrier, carrier aggregation, and multiple-input multiple-output (MIMO) techniques). However, one of the consequences of having to deal with the power amplification of spectrally efficient modulation schemes and techniques (e.g., cyclic prefix orthogonal frequency division multiplexing (OFDM) in 5G New Radio) over wide signal bandwidths and presenting high peak-to-average power ratio (PAPR), is a degradation in the power amplifier (PA) efficiency.

Back-off amplification is the straightforward and power inefficient solution to cope with the linear amplification of modulated signals presenting high PAPR. As an alternative to class-AB linear but power inefficient amplification, more advanced amplification topologies based on dynamic load or dynamic supply modulation have been proposed in the literature. Some of the most popular solutions are envelope tracking PAs [1], Doherty PAs [2,3], load modulated balanced amplifiers (LMBA) [4,5], and outphasing PAs [6,7]. These amplification architectures are oriented at maximizing power efficiency, which means that, in order to guarantee the linearity levels specified in the communications standards, digital predistortion (DPD) linearisation is required.

Active load-modulated PA architectures such as Doherty, LMBA, or outphasing rely on the non-linear interaction between multiple transistors to enhance the average efficiency when operated with modulated signals with high PAPR. In general, for simplicity, these architectures are designed with a single radio frequency (RF) input. However, some benefits can be found by maintaining separate inputs controlled by different upconverter chains. For example, some of the record bandwidth Doherty PAs have separate inputs [8,9], and the advantages of dual-input Doherty compared to single input have been explored in specific studies [10,11,12]. This does not mean that single-input Doherty PAs with good bandwidth do not exist, see for example [13,14]. However, the additional degrees of freedom offered by the separate inputs can be used to optimise the performance on the same or larger bandwidth, or to improve other performance metrics such as linearity and average efficiency [15,16]. Similar considerations can be made for the LMBA, which was originally proposed for telecom applications as a dual-input structure [5]. However, single-input solutions have also been proposed [17], and critically compared to the dual-input case [18]. Furthermore, in this case, the single input solution is viable, with a clear advantage in terms of simplicity, but at the cost of compromised performance. The outphasing represents a very different case, since it requires, in principle, separate inputs that are with constant amplitude and phase modulated only. This is best achieved with separated inputs; however, some attempt has been made to realise single-input narrowband outphasing circuits [19] that demonstrated comparable performance to dual-input cases.

Focusing on dual-input PAs, it is reasonable to state that evaluating the performance of a set of free-parameters often requires experimental cross-validations with significant computational cost and time, especially when the search space is vast. Machine learning strategies were applied to a dual-input Doherty PA in [11] to optimize configuration parameters, such as: bias voltages, input signal phases, and power splitting ratios taking into account a user-defined cost function. Particularizing now for dual-input LMBAs, the effect on linearity of certain configuration parameters (while trying to keep power efficiency values as high as possible) are explored in [20,21]. In [20], for example, a method for predicting the optimum (in terms of linearity) relative phase shift between the two input modulated signals to the LMBA was proposed, assuming certain fixed values for the rest of the free-parameters influencing the linearity versus power efficiency trade-off. Moreover, although the linearisation of LMBAs has been already addressed in the literature, e.g., [18,22], only modulated signals with moderate bandwidths (i.e., several tenths of MHz) have been considered. Nevertheless, in [23], the authors presented the design and linearisation of a LMBA considering several OFDM-based signals with bandwidths up to 200 MHz. With this last challenging bandwidth configuration of 200 MHz, however, the reported adjacent channel leakage ratio (ACLR) after DPD linearisation could not reach the threshold of −45 dBc, while no information on the error vector magnitude (EVM) to quantify the in-band distortion was provided.

Therefore, in this paper, a machine learning assisted auto-tuning approach is proposed to take advantage of the possibilities given by several extra degrees of freedom in LMBAs to enhance the power efficiency figures along wide signal bandwidths (up to 200 MHz instantaneous bandwidth) and high PAPR values (up to 10 dB), while meeting stringent linearity requirements. In order to ensure the best configuration of values of all the free-parameters defining the linearity and power efficiency of the LMBA, a strategy based on optimization techniques is proposed.

In general, the idea behind global optimisation is to find the optimum output value (i.e., the globally best solution in the presence of multiple local optima) of an unknown function with limited evaluations. Several techniques have been proposed in the literature to find the most suitable set of parameters among large tunable ranges. Among the exact methods it is possible to find, for example: Bayesian search algorithms, branch and bound algorithms, adaptive stochastic search methods, or successive approximation methods; while among the heuristic methods, it is possible to find, for example: evolution strategies (e.g., genetic algorithms), the tabu search, or the simulated annealing [24].

In this paper, by using heuristic search algorithms, such as the simulated annealing (SA) or the adaptive Lipschitz optimisation (adaLIPO) selected in this paper for global optimisation purposes, it is possible to find the best configuration for PA biasing, signal calibration, and digital predistortion linearisation that guarantees the linearity specifications, in terms of normalised mean squared error (NMSE), EVM, and ACLR, and maximizes the power efficiency of the LMBA. The proposed methodology for auto-tuning dual-input PAs is general enough to include any particular heuristic search algorithm, DPD behavioural model, or crest factor reduction (CFR) technique. However, a closed-loop adaptive DPD based on a simplified Volterra behavioural model is proposed in this paper. In addition, the orthogonal matching pursuit (OMP) greedy algorithm is used to select the most relevant basis functions necessary to meet the targeted −45 dBc of ACLR.

The remainder of this paper is organized as follows. Section 2 presents a general description of multiple-input PA architectures. Section 3 describes the proposed dual-input PA auto-tuning approach. Section 4 describes the experimental test bench and shows experimental results showing the tuning and linearisation of a LMBA when considering two different OFDM-like test signals with PAPR > 10 dB, of 20 MHz and 200 MHz instantaneous bandwidth, respectively. Finally, the conclusion is given in Section 5.

## 2. Multiple-Input Power Amplifier Architectures

**Figure 1:**
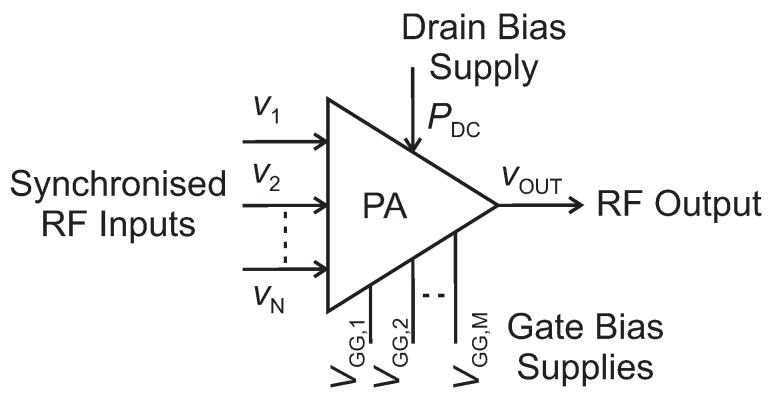
General block diagram of a multiple-input power amplifier.

All the aforementioned PA architectures based on active load modulation (Doherty, LMBA, and outphasing) and separate inputs can be visualised, by generalisation, as the block diagram of Figure 1. The amplifier has *N* RF inputs, a drain or collector bias (or, in some cases, more than one at different rail voltages), and *M* different gate voltages to control the PA stages (e.g., main and auxiliary in a Doherty) independently. A typical example of PA with independent gate voltages is the Doherty, where the main is biased in class AB, and the auxiliary in class C. The instantaneous amplitude and phase of each input, as well as the *M* gate bias voltages, can be controlled and adjusted separately, allowing for a large number of degrees of freedom that can be exploited to optimise a target figure of merit (FOM).

**Figure 2:**
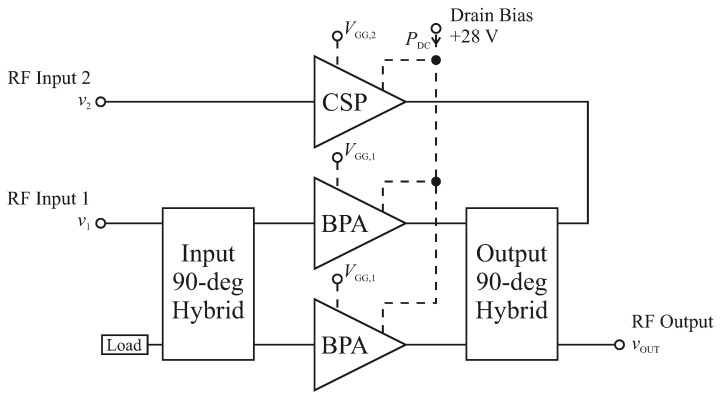
Block diagram of the LMBA used as DUT in this paper.

In this paper, the LMBA presented in [5] is used as device-under-test (DUT), as in [20]. Its block diagram is shown in Figure 2. The LMBA presents two separate RF inputs. The signal $v_{1}$ controls the balanced amplifier (BPA) pair, based on two CGH40025F transistors from Wolfspeed, biased in class AB with $V_{\mathrm{GG},1}$ at −2.8 V corresponding to 80 mA of quiescent drain current. While, the signal $v_{2}$ manages the control signal power (CSP) amplifier, also based on a CGH40025F, and biased in class C, with $V_{\mathrm{GG},2}$, which, as it will be later detailed, it is left as a free parameter within the range of DC voltages −5.5 V to −3.5 V.

In [5], continuous wave (CW), measurements over the 1.7–2.5 GHz frequency range were presented showing maximum power larger than 63 W, and a 8 dB back-off efficiency exceeding 39%. In addition, the linearisation of long-term evolution (LTE) signals with bandwidths of 5 MHz and 20 MHz was also reported. The LMBA configuration considered a manual search for the optimum amplitude, phase, and bias settings. In particular, the relative phase was maintained at a constant offset that led to a good compromise between output power and back-off efficiency, while the relative amplitude was following a square relation between the BPA and CSP inputs [5]. The relative amplitude between the two inputs will now be defined through a shaping function, where it will be possible to tune two degrees of freedom. Similarly, the relative phase will be also a parameter to be optimised. In the following section, a description of the free-parameters that will be left for automatic tuning will be described.

It is worth mentioning that, with respect to the previously reported measurements in [5], in this paper, measurements with modulated signals of several tenths or even hundreds MHz of bandwidth and with PAPRs exceeding 10 dB (e.g., typical PAPRs of carrier aggregated signals around 10 dB or higher) have been considered. Consequently, the average power efficiency shown by the LMBA used as DUT in this paper may be degraded with respect to the values reported in [5]. In addition, a stringent in-band distortion requirement has been considered (i.e., EVM is set to 1%) and thus, the amount of PAPR reduction that can be obtained applying the clipping and filtering CFR technique is limited. Some papers in the literature report higher efficiency figures for Doherty PAs operated under OFDM-based 20 MHz bandwidth signals, but considering unrealistic PAPR values of 6 dB (i.e., the PAPR reduction to achieve the 6 dB does not account for the in-band distortion degradation). Therefore, although the actual DUT used in this paper is not presenting the best power efficiency vs. back-off profile to cope with PAPRs of 10 dB or higher, the auto-tuning methodology proposed in this paper for modulated signals with bandwidths up to 200 MHz, remains valid.

## 3. Description of the Dual-Input PA Auto-Tuning Approach

**Figure 3:**
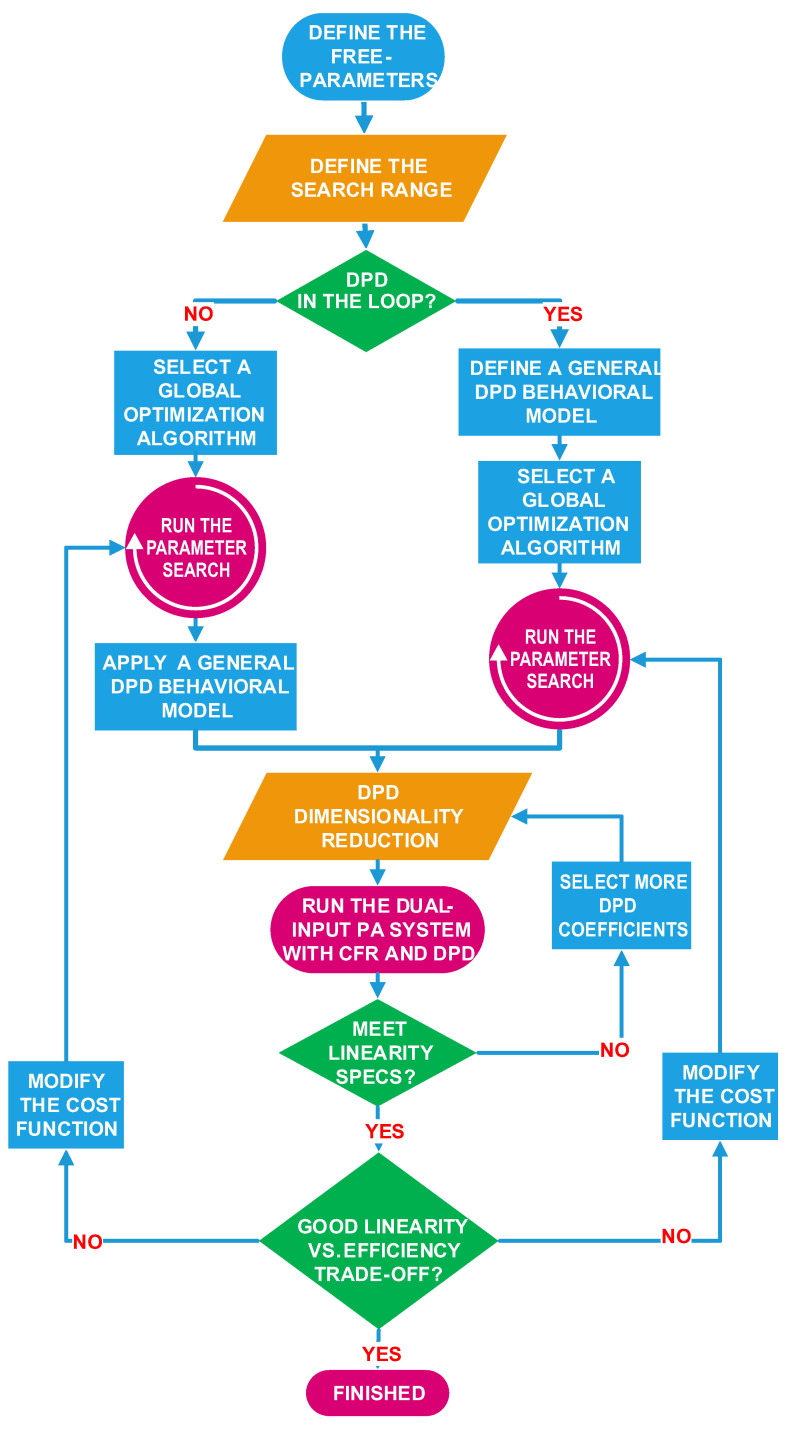
Flowchart of the proposed auto-tuning technique for dual-input PAs.

The tuning approach proposed to configure the dual-input PA is summarized in the flowchart in Figure 3. More specifically, the steps are described in the following:

1. Define the degrees of freedom (free-parameters) to be tuned. Typically, device and system parameters that have an impact on the linearity vs. efficiency trade-off.
2. Define the tuning range of free-parameters (upper and lower bounds). Typically, some preliminary tests, or information about the DUT, is necessary to determine this range.
3. Decide whether to include the DPD in the optimisation process. If included, the final linearity specs can be targeted inside the optimisation algorithm. If not included, lower linearity specs can be targeted, assuming that a later application of DPD will be able to meet system requirements. When considering wideband signals, where the linearity specifications will be more difficult to meet, it is better to include the DPD in the optimisation process. This way, it is possible to avoid solutions where the power efficiency is optimum, but then the linearity levels (mainly in terms of ACLR) cannot be met (even with DPD) without significantly degrading the original power efficiency figures. When including the DPD in the optimisation, the behavioural model needs to be oversized to linearise the dual-input power amplifier under significantly different operation modes. Then, once the optimum configuration is fixed, model order reduction techniques can be applied to the DPD to reduce the number of required parameters.
4. Choose the optimisation algorithm and design the cost (or objective) function. In this cost function, all the FOMs should appear weighted according to their importance. Additionally, some thresholds values for each FOM can also be defined to further penalize not meeting the desired specifications. This is an important feature when dealing with mandatory system requirements such as ACLR limits.
5. Configure the DUT characterisation and capture input–output data searching for the parameters values until the cost function threshold is achieved.
6. Carry out an off-line model order reduction of the DPD behavioural model. A feature selection technique, such as the orthogonal matching pursuit (OMP), is used to reduce the number of parameters of the DPD behavioural model and ensure a well-conditioned estimation.
7. Check the linearity specification after model reduction. If not satisfactory, go back to step 5 and increase the number of coefficients.
8. Check the linearity vs. power efficiency trade-off obtained with the free-parameters found. If not satisfactory, go back to step 4 and redefine the cost function by changing its weights and thresholds.

In the following, a more in depth description of the specific details involving each one of the steps of the proposed tuning approach will be provided.

### 3.1. Free-Parameters of the Dual-Input PA

The DUT used to validate the proposed tuning approach is the LMBA presented in [5].

The figures of merit (FOMs) considered to define the cost or objective function and determine the values of the free-parameters are: the ACLR, the PA power efficiency (η), the NMSE, and the EVM. The out-of-band (defined in terms of ACLR) and the in-band (defined in terms of EVM) linearity specifications must be met (being compliant with the communications standard), while the power efficiency is a figure of merit that justifies the election of one topology in front of another.

**Figure 4:**
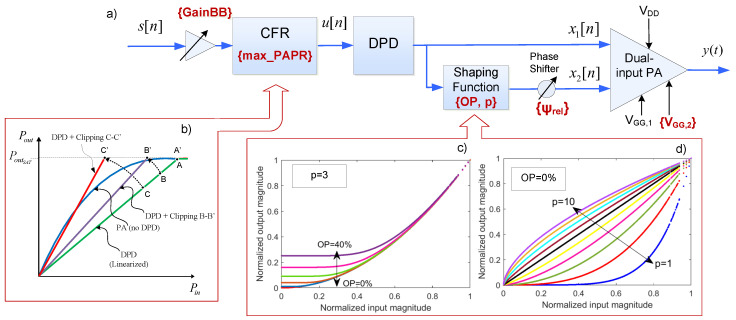
Simplified block-diagram showing the degrees of freedom of the dual-input PA system including DPD and CFR: (**a**) Simplified block diagram of the LMBA transmitter including CFR, DPD, a shaping function and a phase shifter; (**b**) AM-AM characteristics for different baseband gains and clipping values; (**c**) Shaping function input-output characteristics for different values of OP; (**d**) Shaping function input-output characteristics for different values of *p*.

The parameters to be tuned are schematically depicted in Figure 4 and listed in the following:

- Shaping functions parameters; Offset percentage (OP) and degree of the root *p*.
- Relative phase $\left(\psi_{rel}\right)$ between the BPA and CSP signals.
- The DC gate voltage of the CSP amplifier, $V_{\mathrm{GG},2}$.
- The maximum PAPR (max_PAPR) in dB, of the complex baseband signal (u[n]) to be sent.
- The baseband gain (GainBB), which controls the mean input power and thus the input back-off (IBO).

As depicted in Figure 4, by applying some CFR techniques (such as peak cancellation in [25]) it is possible to limit the maximum PAPR. Consequently, the input back-off can be reduced (by increasing GainBB) and thus operate closer to compression, as graphically described in Figure 4b.

The BPA signal is directly defined as $x_{1}\left[n\right]=x\left[n\right]$. While the CSP signal $x_{2}\left[n\right]$ is generated by using a shaping function previously employed in envelope tracking (dynamic supply modulation) and outphasing (dynamic load modulation) applications [26], because it provides two degrees of freedom. As will be described in the following, with one of the parameters it is possible to prevent the signal from dropping to zero, while with the other it is possible to control the shape of input–output characteristic. In particular, following the same approach as in [20], the CSP signal $x_{2}\left[n\right]$ is defined as

(1) $$x_{2}\left[n\right]=x_{sf}\left[n\right]\;e^{i\psi_{rel}}$$

where $\psi_{rel}$ is the relative phase between the BPA and the CSP signals; and the signal after the shaping function $x_{sf}\left[n\right]$ is described as follows,

(2) $$x_{sf}\left[n\right]=A_{s}\left[n\right]\;K_{0}\;e^{i\phi_{x}}$$

with $K_{0}=\frac{max\{|x[n]|\}}{max\{A_{s}\left[n\right]\}}$, $\phi_{x}=phase\left\{x\left[n\right]\right\}$ and where the amplitude relation between the main and control signals is determined as follows,

(3) $$A_{s}\left[n\right]={\left({\left(x_{min}\right)}^{6}+{\left(|x[n]|\right)}^{6}\right)}^{1/p}$$

with *p* being the degree of the root (pth root), and the lower bound $x_{min}$ is defined as

(4) $$x_{min}=max\left\{|x\left[n\right]|\right\}\;OP$$

where OP is the offset percentage, defining the threshold for the detroughing function. The input–output characteristics of the shaping function when sweeping the parameters OP and *p* are depicted in Figure 4c,d.

### 3.2. Digital Predistortion Linearisation

**Figure 5:**
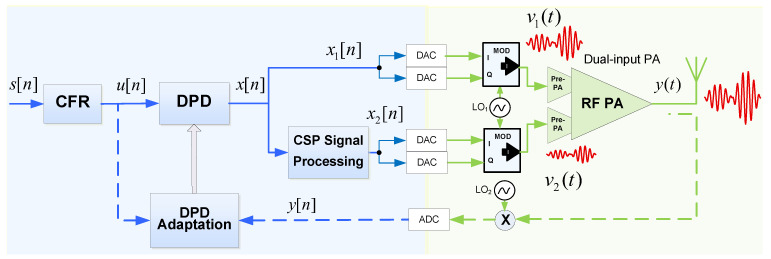
Block diagram of the dual-input PA with CFR and DPD linearisation.

Following the notation of the block diagram in Figure 5, the output of the DPD lineariser can be defined as

(5) x[n]=u[n]−d[n]

where x[n] is the signal at the output of the DPD block, u[n] is the input signal, and d[n] is the distortion signal. In this paper, a simplified Volterra behavioural model is proposed to describe the distortion signal,

(6) $$\begin{array}{c} d\left[n\right]=\sum_{i=0}^{N_{1}-1}\alpha_{i}\;u\left[n-\tau_{i}^{1}\right]+\\ \sum_{j=0}^{N_{3}-1}\sum_{i=0}^{j}\sum_{k=0}^{i}\beta_{kij}\;u\left[n-\tau_{j}^{3}\right]\;u\left[n-\tau_{i}^{3}\right]\;u^{*}\left[n-\tau_{k}^{3}\right]+\\ \sum_{j=0}^{N_{5}-1}\sum_{i=0}^{j}\sum_{k=0}^{i}\sum_{l=0}^{k}\sum_{s=0}^{l}\gamma_{slkij}\;u\left[n-\tau_{j}^{5}\right]\;u\left[n-\tau_{i}^{5}\right]\\ u^{*}\left[n-\tau_{k}^{5}\right]\;u\left[n-\tau_{l}^{5}\right]\;u^{*}\left[n-\tau_{s}^{5}\right]+\\ \sum_{i=0}^{N_{a}-1}\sum_{p=0}^{\frac{P_{a}-7}{2}}\delta_{pi}\;u\left[n-\tau_{i}^{a}\right]\;|u\left[n-\tau_{i}^{a}\right]{|}^{2p+6} \end{array}$$

where $\tau^{1}$, $\tau^{3}$, $\tau^{5}$, and $\tau^{a}$ (with $\tau^{1,3,5,a}\in Z$ and $\tau_{0}^{1,3,5,a}=0$) are the most significant sparse delays of the input (u[n]) that contribute to characterise memory effects. As it can be observed in (6), the first, third and fifth-order Volterra kernels [27] have been included (limiting the number of combinations to avoid repetitions) and for higher odd-order non-linearities, a simple memory polynomial model [28] has been considered. For the linearisation of wideband signals, it is necessary to capture as many cross-memory products as possible, and for that reason, a Volterra-based behavioural model was proposed in this paper.

In a more general and compact notation, (5) can be rewritten as

(7) $$x\left[n\right]=u\left[n\right]-\varphi^{T}\left[n\right]w\left[n\right]$$

where $w\left[n\right]={\left(w_{1}\left[n\right],\cdots ,w_{i}\left[n\right],\cdots ,w_{M}\left[n\right]\right)}^{T}$ is a vector of coefficient at time *n* with dimensions M×1, where *M* is the order of the behavioural model, and $\varphi^{T}\left[n\right]=$ $\left(\varphi_{1}\left[n\right],\cdots ,\varphi_{i}\left[n\right],\cdots ,\varphi_{M}\left[n\right]\right)$ is the vector containing the basis functions $\varphi_{i}\left[n\right]$ (with i=1,⋯,M) following the Volterra-based model in (6). The mapping between the simplified Volterra-based model specific coefficients ($\alpha_{i},\beta_{kij},\gamma_{slkij}$ and $\delta_{pi}$) in (6) and the general purpose DPD coefficients $w_{i}\left[n\right]$ in (7) is straightforward.

Considering now a general matrix notation, (5) can be rewritten as

(8) x=u−Uw

where $x={\left(x\left[0\right],\cdots ,x\left[n\right],\cdots ,x\left[N-1\right]\right)}^{T}$ and $u={\left(u\left[0\right],\cdots ,u\left[n\right],\cdots ,u\left[N-1\right]\right)}^{T}$, with n=0,⋯,N−1, are the predistorted and input vectors, respectively, and U=${\left(\varphi \left[0\right],\cdots ,\varphi \left[n\right],\cdots ,\varphi \left[N-1\right]\right)}^{T}$ is the N×M data matrix, with *N* being the number of samples and *M* being the number of basis functions or the order of the model.

In order to extract the DPD coefficients iteratively, a direct learning approach [29,30] is adopted. At the ith iteration, the coefficients are calculated solving the least squares (LS) problem as follows:

(9) $$w^{i+1}=w^{i}+\mu {\left(U^{H}U\right)}^{-1}U^{H}e$$

with μ(0≤μ≤1) being the weighting factor and $e={\left(e\left[0\right],\cdots ,e\left[n\right],\cdots ,e\left[N-1\right]\right)}^{T}$ is the N×1 vector of the error defined as

(10) $$e=\frac{y}{G_{0}}-u$$

where $G_{0}$ determines the desired linear gain of the PA, and where y and u are the N×1 vectors of the PA output and the transmitted input, respectively.

In addition, in order to further simplify the number of basis functions defining the proposed DPD behavioural model, it is possible to apply feature selection techniques. One popular solution in the field of DPD linearisation for reducing the number of required coefficients of the DPD function in the forward path is the orthogonal matching pursuit (OMP) greedy algorithm [31,32].

### 3.3. Global Optimisation Algorithms

In this paper, the use of two heuristic search algorithms to determine the free-parameters of the dual-input PA have been considered, namely, the well-known simulated annealing and the adaLIPO algorithm. A brief description of both is given in the following.

#### 3.3.1. Simulated Annealing

One of the most famous large scale heuristic searching methods is simulated annealing, which was first introduced by Kirkpatrick in 1983 [33]. The SA method (named after a technique in metallurgy involving heating and controlled cooling of a material to increase the size of its crystals and reduce their defects) performs well in the case of large scale searching, and also has a good property of converging. Following the analogy with metallurgy, the slow cooling in the simulated annealing has to do with a slow decrease in the probability of accepting worse solutions as the solution space is explored. To find the global optimum solution, the algorithm has to be able to carry out an extensive search, and that is the reason why accepting worse solutions is a fundamental property. Therefore, at each iteration, the algorithm randomly selects a solution and evaluates it, then decides the next move based on either one of two probabilities according to the quality of the new solution in comparison to the previous ones. During the search, the SA parameter named temperature (again, in analogy with metallurgy) is progressively decreased (until reaching the zero value), and the probabilities of moving to a better new solution and moving to a worse new solution updated accordingly.

#### 3.3.2. Adaptive Lipschitz Optimisation

The smoothness-based approach to global optimisation assumes that the system presents some regularity with respect to the input. In particular, the use of the Lipschitz constant (the bound of the first derivative of a Lipschitz function, i.e., a continuous function limited in how fast it can change) in [34,35], played a key role in the development of many efficient global optimisation algorithms. The adaLIPO algorithm proposed in [36] is oriented to exploit the global smoothness of the unknown function for global optimisation and, according to the authors, can achieve faster rates of convergence on globally smooth problems than the previously known methods.

**Figure 6:**
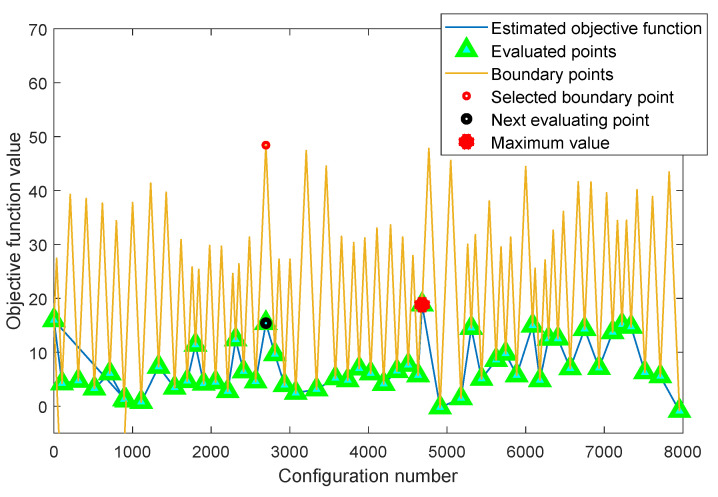
Graphical example of the adaLIPO algorithm.

The principles of the adaLIPO algorithm are summarized in the example depicted in Figure 6, consisting in the optimisation of a one-dimensional function. The blue line is the given function to be optimised. The basics of the adaLIPO algorithm consist in maintaining a piecewise upper bound of the given function to be optimised, according to the Lipschitz constant. At the beginning, for a few iterations, the Lipschitz constant and the bound (the orange line) are estimated. Then, as shown in Figure 6, the algorithm finds the maximum value of the upper bound (the red circle) and obtains its real value (the black circle) after the evaluation of the given function. In the next iteration, the Lipschitz constant and the bound will be updated, and using a Bernoulli distribution, among all cross points of the upper bound, the next point will be selected. The optimising procedure will converge after several iterations.

## 4. Experimental Setup and Results

### 4.1. Experimental Testbench

**Figure 7:**
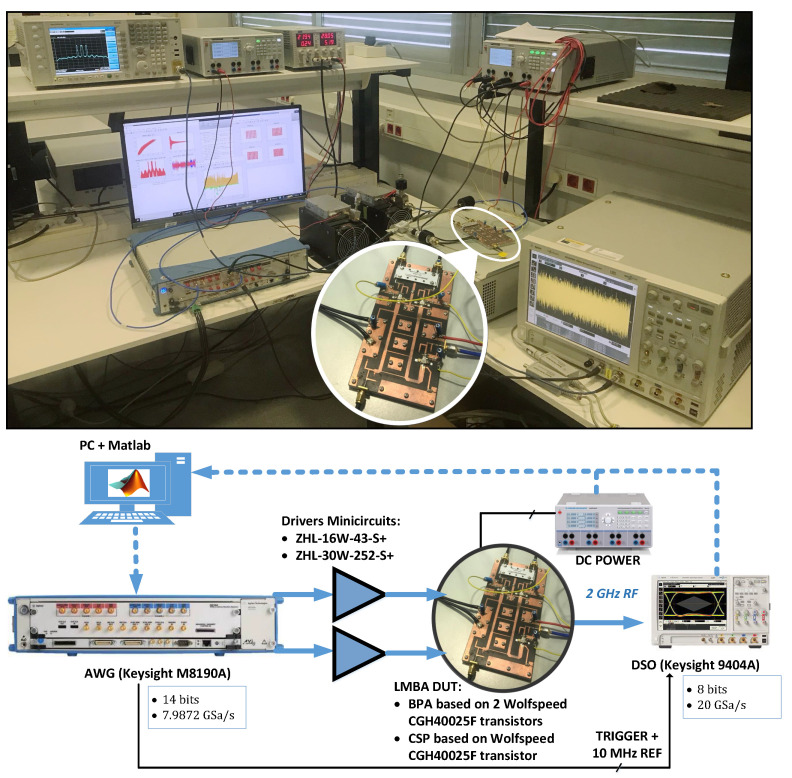
Picture of the test setup employed for experimental validation.

The dual-input PA system was experimentally evaluated using a Matlab-controlled digital linearisation test bench, as shown in Figure 7, interfacing waveform generation and acquisition instruments. In order to account for the out-of-band distortion, a 614.4 MSa/s DPD signal was digitally up-converted to the 2 GHz RF frequency and digital to analogue converted (through the arbitrary waveform generator (AWG) M8190A from Keysight Technologies, Santa Rosa, CA, USA, with a clock rate of 7.9872 GHz and 14 bits) to feed the dual-input PA. The PA output signal was attenuated, RF sampled with the digital storage oscilloscope (DSO) 90404A from Keysight Technologies at 20 GSa/s with 8-bit resolution (applying averages to reduce the noise floor), digital down-converted, and resampled for time-alignment and DPD processing. A N9020A MXA signal analyzer from Keysight Technologies was used to characterise the spectrum at the output of the PA.

### 4.2. General Considerations

The proposed auto-tuning approach for LMBA or dual-input PA systems was tested with OFDM-like (LTE) waveforms. In particular, two types of test signals were considered: (i) a 64 QAM modulated 20 MHz bandwidth LTE signal (LTE-20) at 2 GHz RF frequency with 10.2 dB of PAPR, and (ii) a non contiguous intra-band carrier-aggregated (CA) LTE system consisting in 4 channels of 64 QAM modulated LTE-20 signals (CA-4 × LTE-20) spread in 200 MHz instantaneous bandwidth at 2 GHz RF frequency and a PAPR of 10.7 dB. To be noted, these signals are more demanding than the ones previously used to characterise the same DUT [5]. For each signal, the training data set consisted in 307,200 complex-valued data samples, which, considering a baseband clock of 614.4 MSa/s, corresponded to 0.5 mseconds of an OFDM waveform (i.e., approximately 8 OFDM symbols in LTE). The obtained LMBA configuration was later validated (including DPD linearisation) considering different batches of data of 307,200 complex-valued data samples. Therefore, different sets of data were used for training and validation.

**Figure 8:**
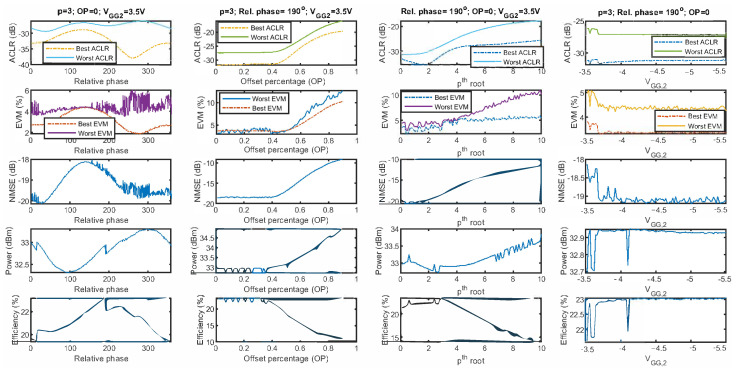
Evaluation of the FOMs, namely: best and worst case, EVM (best and worst case (ACLR)), NMSE, mean output power, mean power efficiency; when sweeping the values of the following degrees of freedom individually: relative phase ($\psi_{rel}$), offset percentage (OP), degree of the root (*p*), and the gate voltage of the CSP amplifier ($V_{\mathrm{GG},2}$).

Following the proposed procedure schematically described in Figure 3, the first step is to define the free-parameters to be optimised. In order to show the difficulty of properly tuning the parameters defined in Section 3.1, Figure 8 shows the evolution of the best-case and worst-case ACLR, best-case and worst-case EVM, the NMSE, the output power, and the power efficiency for a 200 MHz CA-4 × LTE-20 test signal, when:

- Sweeping the relative phase ($\psi_{rel}$), but keeping p=3, OP=0, and $V_{\mathrm{GG},2}=-3.5$ V.
- Sweeping the OP, but keeping p=3, $\psi_{rel}=190^{\circ }$, and $V_{\mathrm{GG},2}=-3.5$ V.
- Sweeping *p*, but keeping, OP=0, $\psi_{rel}=190^{\circ }$, and $V_{\mathrm{GG},2}=-3.5$ V.
- Sweeping $V_{\mathrm{GG},2}$, but keeping p=3, OP=0, and $\psi_{rel}=190^{\circ }$.

For simplicity, no CFR has been considered, and the input gain has been kept fixed. As observed in Figure 8, by sweeping the values of one parameter and fixing the values of the rest, it is possible to evaluate the different FOMs and determine the best configuration for each one individually. However, by fixing some of their values, the search space is being constrained and thus, there is no guarantee that the solution found for this specific set of parameters is a global optimum.

Therefore, to properly tune the free-parameters, the next step, according to the fluxogram in Figure 3, is to define the search range (upper and lower bounds). This is empirically determined and, in this paper, the same search range was considered for both test cases. The upper and lower bounds defined for the free-parameters under search were:

- Offset percentage, OP=[0.01,0.40]. It was empirically found (as an example, see Figure 8) that for OP>0.4, the linearity and efficiency performance was significantly degraded.
- degree of the root, p=[1.0,10.0]. It was empirically found that for p>10, no significant variations are appreciated in the linearity performance.
- Relative phase, $\psi_{rel}={\left[0,\;359\right]}^{o}$.
- The gate voltage of the CSP amplifier, $V_{\mathrm{GG},2}=\left[-3.5,\;-5.5\right]V$. This provides a reasonable variation between a deep-class C condition that should favour efficiency, and a near-class B bias where linearity should improve.
- The maximum PAPR, max_PAPR=[7.0,12.0]dB. For PAPR values lower than 7 dB, the EVM degradation result was unacceptably high, while no CFR was applied for PAPR values higher than 11.5 dB.
- The baseband gain, GainBB=[16.0,19.0]. This range of baseband gain values provides a variation of 1.5 dB to adjust the IBO.

At this point, it is necessary to decide: (i) if the DPD linearisation needs to be included in the search procedure, (ii) the optimisation algorithm to be used, and (iii) the FOMs, weights, and thresholds of the cost function. As it will be shown in the next subsections, DPD linearisation was included in the search process only for the CA-4 × LTE-20 test signal case. In addition, despite the fact that in both test cases the use of both SA and adaLIPO algorithms to determine the values of the free-parameters were considered, the cost functions used in each test-case were different.

### 4.3. Test Case 1: 20 MHz Bandwidth LTE Signal (LTE-20)

To start with, for the 20 MHz LTE case, the DPD was not included in the optimisation algorithm. The cost function for the LTE-20 case is defined in (11),

(11) $$\begin{array}{c} J=\left(\eta_{th}-\eta \right)\lambda_{\mathrm{eff}}+\left(\mathrm{ACLR}-{\mathrm{ACLR}}_{th}\right)\lambda_{\mathrm{ACLR}}\\ +\left(\mathrm{NMSE}-{\mathrm{NMSE}}_{th}\right)\lambda_{\mathrm{NMSE}}+\left(\mathrm{EVM}-{\mathrm{EVM}}_{th}\right)\lambda_{\mathrm{EVM}} \end{array}$$

As depicted in Table 1, with this configuration of weights, more importance is given to minimize the out-of-band distortion (i.e., ACLR) and maximize power efficiency, while the in-band distortion (i.e., NMSE and EVM) requirements are more relaxed, since they are easier to meet.

**Figure 9:**
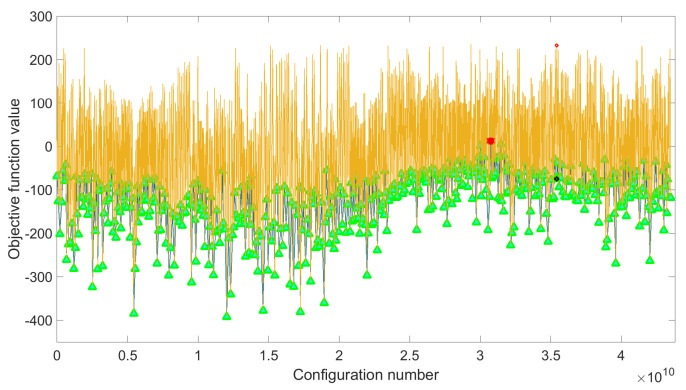
Example of the adaLIPO search for the LTE-20 signal test case.

The results obtained when considering both SA and adaLIPO optimisations are listed in Table 1. As an example, Figure 9 shows the solution found by the adaLIPO algorithm (out of $4.3507\times 10^{10}$ possible configurations) for the given objective or cost function. Note that the adaLIPO algorithm searches the maximum of the cost function, consequently, the sign of the cost function described in (11) has to be changed to run the algorithm. In addition, taking into account that the weights of the cost function are multiplying the FOMs, the threshold values defined in this cost function have no real impact or penalization effect. In this particular case, they are simply included to create an offset for better interpreting the score value (i.e., positive score values correspond to configurations where most of the targeted thresholds are met).

**Figure 10:**
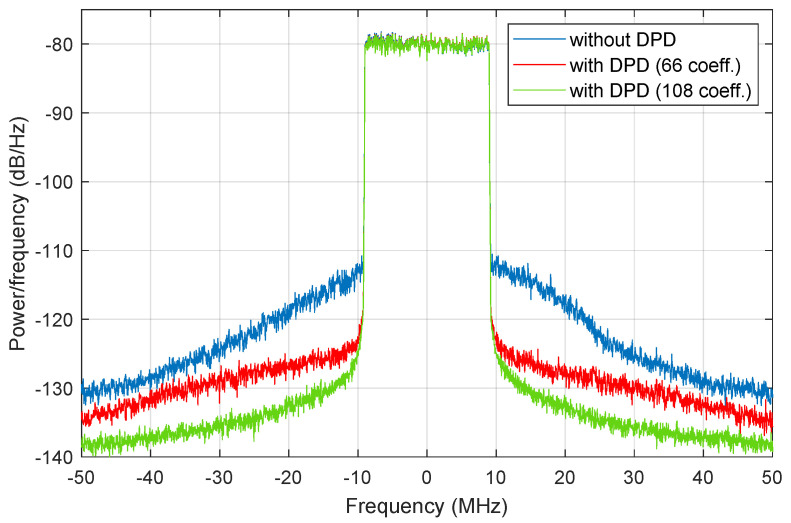
LTE-20 signal test case. Output power spectra before and after DPD linearisation, when considering a DPD behavioural model with 66 and 108 coefficients, respectively, and taking into account the adaLIPO parameters configuration.

With the free-parameters found in Table 1 using both SA and adaLIPO optimisation algorithms, DPD linearisation was applied, obtaining the results listed in Table 2. To be noted, no CFR was applied, since both algorithms discard to apply CFR reduction. In addition, in Table 2, a triple compromise can be observed among the power efficiency, the linearity, and the computational complexity. The power efficiency is around 31% (with less than 1 percentage point of variation) independently on the optimisation method or the number of coefficients of the DPD, since the PA power efficiency is more sensitive to the chosen input power back-off. The linearity levels are easily met (e.g., the EVM after DPD is always below 1%), but it is possible to trade-off the ACLR levels and the number of coefficients by using a dimensionality reduction method such as the OMP algorithm. Therefore, as depicted in Figure 10 and listed in Table 2, the ACLR specifications (i.e., ACLR<−45 dBc) can be met with only 66 coefficients or, alternatively, achieving better spectral regrowth compensation by including more DPD coefficients (e.g., up to 108 coefficients) when considering the parameters configuration found by the adaLIPO algorithm and listed in Table 1.

### 4.4. Test Case 2: 200 MHz Bandwidth CA-4 × LTE-20 Signal

For the CA-4 × LTE-20 test signal case, the attempts of optimisation without DPD inclusion in the process did not lead to a configuration where the output signal was compliant with the ACLR and EVM thresholds (even when applying DPD linearisation a posteriori, i.e., once the optimum configuration was found). Therefore, DPD linearisation was included to run the optimisation search process. It was important to make sure that the solution found resulted in a PA behaviour that could be later linearised with the 200 MHz instantaneous bandwidth signal. A general Volterra-like behavioural model (as described in (6)) was considered with a generic configuration that yielded to a DPD behavioural model with 592 coefficients. Another change compared to the 20 MHz LTE case was related to the definition of the cost function, where some thresholds were added together with the weights (this time defined as exponents) to not only emphasize the desired behaviour, but also to add further penalization in case of not meeting the linearity threshold values.

(12) $$\begin{array}{c} J={\left(\eta_{th}-\eta \right)}^{\lambda_{\mathrm{eff}}}+{\left(\mathrm{ACLR}-{\mathrm{ACLR}}_{th}\right)}^{\lambda_{\mathrm{ACLR}}}\\ +{\left(\mathrm{NMSE}-{\mathrm{NMSE}}_{th}\right)}^{\lambda_{\mathrm{NMSE}}}+{\left(\mathrm{EVM}-{\mathrm{EVM}}_{th}\right)}^{\lambda_{\mathrm{EVM}}} \end{array}$$

**Figure 11:**
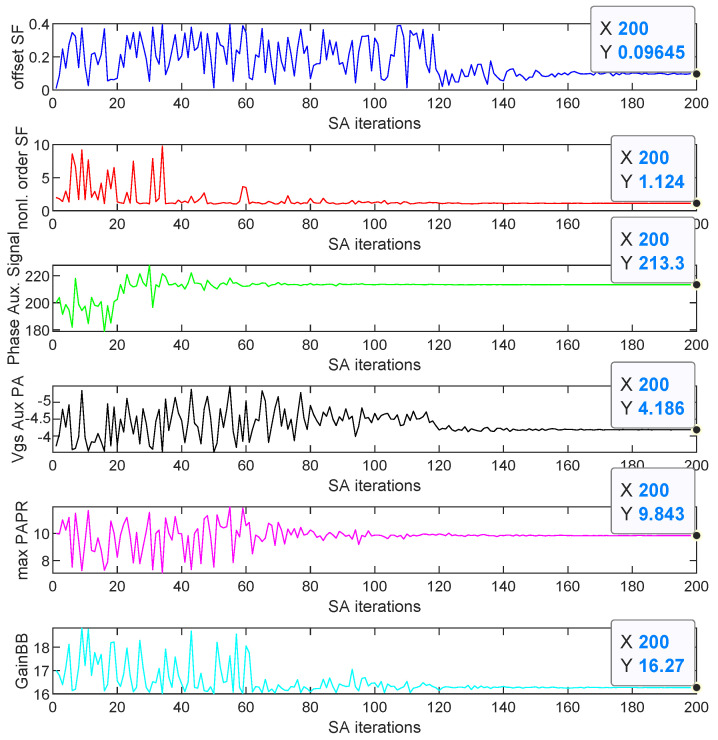
Evolution of the parameter values for different SA iterations for the CA-4 × LTE-20 signal test case.

**Figure 12:**
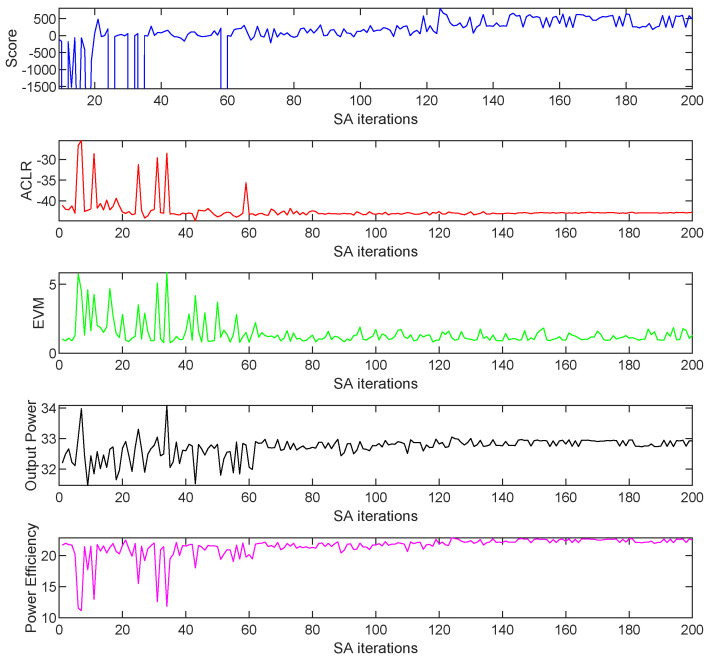
Evolution of the FOMs along different SA iterations for the CA-4 × LTE-20 signal test case.

The results obtained when considering both SA and adaLIPO optimisations including DPD are listed in Table 3. As an example, Figure 11 and Figure 12 show the evolution of free-parameter values and the evolution of the FOMs, respectively, along 200 SA iterations. The values to which the free-parameters converged are shown in Figure 11 and listed in Table 3.

With the free-parameters found in Table 3 using both SA and adaLIPO optimisation algorithms, defined by the maxPAPR parameter (CFR) and using the Volterra-based DPD model in (6) (DPD) were applied, obtaining the results listed in Table 4 and showing the linearity vs. efficiency trade-off. As it can be observed, even when the parameters configuration differ between SA and adaLIPO, their performance is quite similar. For the 200 MHz instantaneous bandwidth signal tested, the out-of-band and in-band linearity specifications can be met with a mean output power around 33 dBm, and a power efficiency around 22%.

**Figure 13:**
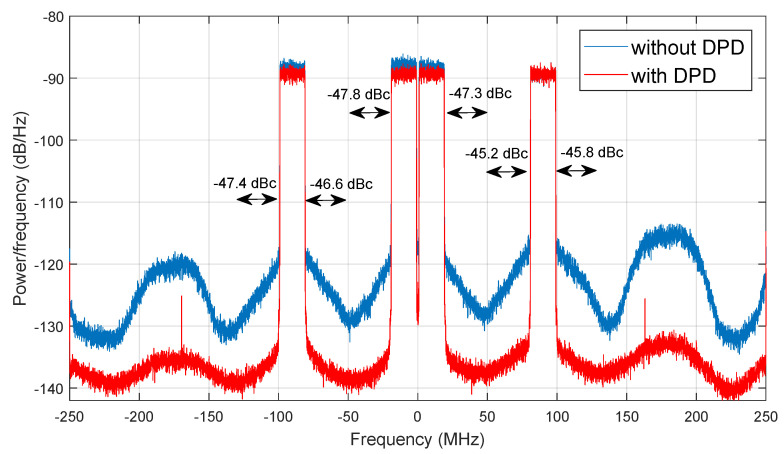
Output power spectra before and after DPD linearisation for the CA-4 × LTE-20 signal test case.

**Figure 14:**
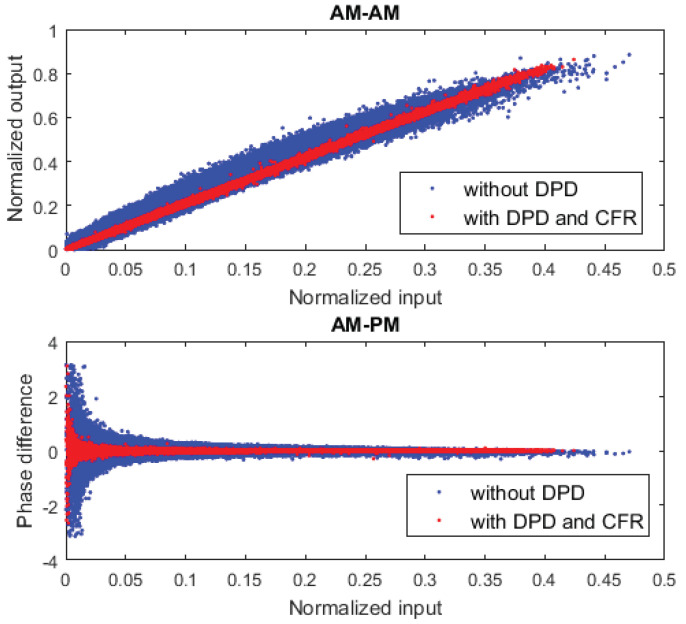
AM-AM and AM-PA before and after DPD for the CA-4 × LTE-20 signal test case.

In addition, after applying the OMP algorithm for feature selection, it was possible to reduce the number of coefficients of the DPD behavioural model up to 374 coefficients in the case of the SA configuration, and 364 coefficients in the case of the adaLIPO configuration, and still be compliant with the required linearity specifications. Figure 13 shows the spectra of the 200 MHz instantaneous bandwidth CA-4 × LTE-20 test signal before and after DPD linearisation (considering the SA configuration in Table 4); while Figure 14 depicts the AM-AM and AM-PM characteristics before and after DPD linearisation. Note that in both cases, CFR was applied to the original signal to limit the PAPR to 9.8 dB (as described in Table 3).

## 5. Conclusions

In this paper, an approach to exploit at best dual-input PAs in terms of maximizing power efficiency along wide bandwidths while being compliant with the linearity specifications was proposed. The proposed technique relies on conducting a global optimisation to find the optimum values of a set of key circuit and system level parameters that properly combined with DPD linearisation and CFR techniques can find a good compromise for the inherent linearity vs. efficiency trade-off.

The proposed approach has been validated through experimental results. In this paper, the SA and adaLIPO heuristic search global optimisation algorithms were used to find the best parameter configuration, taking into account two different test cases with different cost functions for each one. By using a LMBA and after properly tuning the selected free-parameters, it was possible to achieve power efficiency values greater than 30% when considering the LTE-20 test signal. Moreover, up to 22% of mean power efficiency was obtained when considering the CA-4 × LTE-20 test signal with 200 MHz instantaneous bandwidth. In both test-cases, the peak-to-average power ratio (PAPR) of the signals was greater than 10 dB, the out-of-band linearity requirements (ACLR<−45 dBc) were met, and the error vector magnitude was kept always below 1%.

Despite obtaining different parameters’ configurations depending on the type of heuristic search algorithm used, in both test cases (i.e., LTE-20 and CA-4 × LTE-20 test cases) the linearisation performance (in terms of ACLR and EVM) and power efficiency figures obtained were quite similar independently of the optimisation algorithm used.

The LMBA used in this paper was not designed to present optimum efficiency for such large PAPR values, therefore the efficiency figures obtained are not very high. However, it has been used as a vehicle to successfully demonstrate that the proposed auto-tuning methodology (including CFR and DPD linearisation) is valid for addressing the inherent linearity versus power efficiency trade-off in dual-input PAs.

## Figures and Tables

**Table 1:** Parameters configuration for a LTE 20 MHz bandwidth signal.

Opt. Config.	Threshold	Weight	Optim. Values
			OP=0.31
Simulated			p=6
Annealing			$\psi_{rel}=245$°
(No DPD)			maxPAPR=10.8 dB
	η=25%	$\lambda_{\mathrm{eff}}=10$	$V_{\mathrm{GG},1}=-5.4$ V
	ACLR = −45 dBc	$\lambda_{\mathrm{ACLR}}=7$	GainBB=6.0
	NSME = −26 dB	$\lambda_{\mathrm{NMSE}}=2$	OP=0.26
	EVM = 4%	$\lambda_{\mathrm{EVM}}=5$	p=4.6
adaLIPO			$\psi_{rel}=254$°
(No DPD)			maxPAPR=11 dB
			$V_{\mathrm{GG},1}=-5.3$ V
			GainBB=6.1

**Table 2:** Linearisation results with the LTE 20 MHz bandwidth signal.

Opt. Config.	Worst ACLR	NSME	Worst EVM	Optput Power	η
	(dBc)	(dB)	(%)	(dBm)	(%)
SA config.					
without DPD	−38.7	−29.0	2.0	36.6	31.1
SA config.					
with 108 coeff. (DPD)	−49.0	−37.6	0.8	36.6	30.3
SA config.					
with 62 coeff. (DPD)	−48.3	−37.8	0.7	36.4	30.7
adaLIPO config.					
without DPD	−36.7	−27.5	2.3	36.2	31.0
adaLIPO config.					
with 108 coeff. (DPD)	−53.4	−40.9	0.6	36.2	31.5
adaLIPO config.					
with 66 coeff. (DPD)	−46.7	−38.5	0.7	36.2	31.8

**Table 3:** Parameters configuration for the CA-4 × LTE-20 200 MHz bandwidth signal.

Opt. Config.	Thresholds	Weight	Optim. Values
			OP=0.09
			p=1.12
Simulated			$\psi_{rel}=213$°
Annealing			maxPAPR=9.8 dB
(with DPD)	η = 19%	$\lambda_{\mathrm{eff}}=5$	$V_{\mathrm{GG},1}=-4.2$ V
	ACLR = −45 dBc	$\lambda_{\mathrm{ACLR}}=5$	GainBB=16.3
	NSME = −30 dB	$\lambda_{\mathrm{NMSE}}=1$	OP=0.02
	EVM = 1%	$\lambda_{\mathrm{EVM}}=1$	p=1.5
adaLIPO			$\psi_{rel}=182$°
(with DPD)			maxPAPR=9.6 dB
			$V_{\mathrm{GG},1}=-4.6$ V
			GainBB=16.3

**Table 4:** Linearisation results with the CA-4 × LTE-20 200 MHz bandwidth signal.

Opt. Config.	Worst ACLR	NSME	Worst EVM	Optput Power	η
	(dBc)	(dB)	(%)	(dBm)	(%)
SA with CFR and					
without DPD	−30.3	−20.0	4.1	33.8	24.8
SA with CFR and					
with DPD	−45.2	−35.8	0.9	32.9	22.2
adaLIPO with CFR and					
without DPD	−30.4	−20.1	4.3	33.7	24.7
adaLIPO with CFR and					
with DPD	−45.1	−35.5	0.9	32.8	22.2

## Data Availability

The data presented in this study are available on reasonable request from the corresponding author.
